# Determinants of Physical Activity among Patients with Colorectal Cancer: From Diagnosis to 5 Years after Diagnosis

**DOI:** 10.1249/MSS.0000000000003351

**Published:** 2023-11-27

**Authors:** KAREL C. SMIT, JEROEN W.G. DERKSEN, REBECCA K. STELLATO, ANNE-SOPHIE VAN LANEN, EVERTINE WESSELINK, ERIC J. TH. BELT, MARISSA CLOOS-VAN BALEN, PETER PAUL L.O. COENE, JAN WILLEM T. DEKKER, JAN WILLEM DE GROOT, ANNEBETH W. HARINGHUIZEN, HENK K. VAN HALTEREN, TJARDA T. VAN HEEK, HELGI H. HELGASON, MATHIJS P. HENDRIKS, IGNACE H.J.T. DE HINGH, RONALD HOEKSTRA, DANNY HOUTSMA, JOHAN J.B. JANSSEN, NIELS KOK, JOOP L.M. KONSTEN, MAARTJE LOS, MARTIJN R. MEIJERINK, LEONIE J.M. MEKENKAMP, KOEN C.M.J. PEETERS, MARCO B. POLÉE, RON C. RIETBROEK, ANANDI H.W. SCHIPHORST, RUUD W.M. SCHRAUWEN, JENNIFER SCHREINEMAKERS, MARK P.S. SIE, LIEKE SIMKENS, ERIC J.A. SONNEVELD, FREDERIEK TERHEGGEN, LISELOT VALKENBURG-VAN IERSEL, WOUTER J. VLES, DARIA K. WASOWICZ-KEMPS, JOHANNES H.W. DE WILT, DIEUWERTJE E. KOK, RENATE M. WINKELS, ELLEN KAMPMAN, FRÄNZEL J.B. VAN DUIJNHOVEN, MIRIAM KOOPMAN, ANNE M. MAY

**Affiliations:** 1 Department of Epidemiology, Julius Center for Health Sciences and Primary Care, University Medical Center Utrecht, Utrecht University, Utrecht, THE NETHERLANDS; 2 Department of Data Science and Biostatistics, Julius Center for Health Sciences and Primary Care, University Medical Center Utrecht, Utrecht University, Utrecht, THE NETHERLANDS; 3 Division of Human Nutrition and Health, Wageningen University & Research, Wageningen, THE NETHERLANDS; 4 Department of Surgery, Albert Schweitzer Hospital, Dordrecht, THE NETHERLANDS; 5 Department of Medical Oncology, Groene Hart Hospital, Gouda, THE NETHERLANDS; 6 Department of Surgery, Maasstad Hospital, Rotterdam, THE NETHERLANDS; 7 Department of Surgery, Reinier de Graaf Hospital, Delft, THE NETHERLANDS; 8 Department of Medical Oncology, Isala Hospital, Zwolle, THE NETHERLANDS; 9 Department of Medical Oncology, Ziekenhuis Gelderse Vallei, Ede, THE NETHERLANDS; 10 Department of Medical Oncology, Admiraal de Ruyter Hospital, Goes, THE NETHERLANDS; 11 Department of Surgery, Ziekenhuis Gelderse Vallei, Ede, THE NETHERLANDS; 12 Department of Medical Oncology, Haaglanden Medical Center, Den Haag, THE NETHERLANDS; 13 Department of Medical Oncology, Northwest Clinics, Alkmaar, THE NETHERLANDS; 14 Department of Surgery, Catharina Hospital, Eindhoven, THE NETHERLANDS; 15 Department of Medical Oncology, Ziekenhuisgroep Twente, Hengelo, THE NETHERLANDS; 16 Department of Medical Oncology, Haga Hospital, Den Haag, THE NETHERLANDS; 17 Department of Medical Oncology, Canisius Wilhelmina Hospital, Nijmegen, THE NETHERLANDS; 18 Department of Gastrointestinal Oncology, Antoni Van Leeuwenhoek-Netherlands Cancer Institute, Amsterdam, THE NETHERLANDS; 19 Department of Surgery, Viecuri Hospital, Venlo, THE NETHERLANDS; 20 Department of Medical Oncology, St. Antonius Hospital, Nieuwegein, THE NETHERLANDS; 21 Department of Radiology and Nuclear Medicine, VU Medical Center, Amsterdam, THE NETHERLANDS; 22 Department of Medical Oncology, Medisch Spectrum Twente, Enschede, THE NETHERLANDS; 23 Department of Surgery, Leiden University Medical Center, University of Leiden, Leiden, THE NETHERLANDS; 24 Department of Medical Oncology, Medical Center Leeuwarden, Leeuwarden, THE NETHERLANDS; 25 Department of Medical Oncology, Rode Kruis Hospital, Beverwijk, THE NETHERLANDS; 26 Department of Surgery, Diakonessenhuis Hospital, Utrecht, THE NETHERLANDS; 27 Department of Gastroenterology and Hepatology, Bernhoven Hospital, Uden, THE NETHERLANDS; 28 Department of Surgery, Amphia Hospital, Breda, THE NETHERLANDS; 29 Department of Medical Oncology, ZorgSaam Hospital, Terneuzen, THE NETHERLANDS; 30 Department of Medical Oncology, Maxima Medical Center, Eindhoven, THE NETHERLANDS; 31 Department of Surgery, Dijklander Hospital, Hoorn, THE NETHERLANDS; 32 Department of Medical Oncology, Bravis Hospital, Roosendaal, THE NETHERLANDS; 33 Maastricht University Medical Center, Department of Internal Medicine, Division of Medical Oncology, GROW, Maastricht University, Maastricht, THE NETHERLANDS; 34 Department of Surgery, Ikazia Hospital, Rotterdam, THE NETHERLANDS; 35 Department of Surgery, Elisabeth-TweeSteden Hospital, Tilburg, THE NETHERLANDS; 36 Department of Surgery, Radboud University Medical Center, University of Nijmegen, Nijmegen, THE NETHERLANDS; 37 Department of Medical Oncology, University Medical Center Utrecht, Utrecht University, Utrecht, THE NETHERLANDS

**Keywords:** COLORECTAL NEOPLASMS, GUIDELINES, HEALTH BEHAVIOR, SURVIVORSHIP, SPORT PARTICIPATION

## Abstract

**Introduction:**

Physical activity (PA) is associated with higher quality of life and probably better prognosis among colorectal cancer (CRC) patients. This study focuses on determinants of PA among CRC patients from diagnosis until 5 yr postdiagnosis.

**Methods:**

Sociodemographic and disease-related factors of participants of two large CRC cohort studies were combined. Moderate-to-vigorous PA during sport and leisure time (MVPA-SL) was measured at diagnosis (T0) and 6, 12, 24, and 60 months (T6 to T60) postdiagnosis, using the SQUASH questionnaire. Mixed-effects models were performed to identify sociodemographic and disease-related determinants of MVPA-SL, separately for stage I–III colon (CC), stage I–III rectal cancer (RC), and stage IV CRC (T0 and T6 only). Associations were defined as consistently present when significant at ≥4 timepoints for the stage I–III subsets. MVPA-SL levels were compared with an age- and sex-matched sample of the general Dutch population.

**Results:**

In total, 2905 CC, 1459 RC and 436 stage IV CRC patients were included. Patients with higher fatigue scores, and women compared with men had consistently lower MVPA-SL levels over time, regardless of tumor type and stage. At T6, having a stoma was significantly associated with lower MVPA-SL among stage I-III RC patients. Systemic therapy and radiotherapy were not significantly associated with MVPA-SL changes at T6. Compared with the general population, MVPA-SL levels of CRC patients were lower at all timepoints, most notably at T6.

**Conclusions:**

Female sex and higher fatigue scores were consistent determinants of lower MVPA-SL levels among all CRC patients, and MVPA-SL levels were lowest at 6 months postdiagnosis. Our results can inform the design of intervention studies aimed at improving PA, and guide healthcare professionals in optimizing individualized support.

The number of colorectal cancer (CRC) survivors experiencing possible symptoms related to both the disease and its treatment is increasing due to a high incidence of CRC, a growing number of treatment options and gradual improvement of patient outcomes including survival (1,2). Commonly reported short-term symptoms include body image distress, bowel dysfunction, peripheral neuropathy, fatigue, psychological complaints, and insomnia (3). Common long-term symptoms include peripheral neuropathy, fatigue, and diarrhea (4).

Physical activity (PA) is known to have beneficial effects on several symptoms among CRC patients both during and after treatment. Beneficial effects include a reduction of fatigue and anxiety and depression symptoms, and improvement of health-related quality of life (HRQoL) (5,6). On the other hand, a CRC diagnosis may negatively impact PA levels. Prior studies showed that CRC patients may be less active after diagnosis compared with before diagnosis (7). Moreover, around half of the general population already fails to meet PA guidelines and this proportion is likely even higher in patients diagnosed with CRC (8,9).

Several studies have investigated determinants related to habitual PA levels among CRC patients, and found associations with both sociodemographic determinants, such as age and disease-related determinants, including the presence of a stoma (9–14). However, results varied between studies, possibly due to variation in study design, type of PA questionnaires used, PA outcome categorization, and range of determinants included. To date, most studies analyzed only one point in time and did not assess determinants of PA over time. Moreover, many studies only focused on long-term CRC survivors and PA levels from diagnosis onwards were unknown. Therefore these studies did not investigate determinants of PA during diagnosis and treatment. In addition, most studies did not include, or had a limited sample of, patients with stage IV disease, and none examined associations for rectal cancer (RC) and colon cancer (CC) patients separately. Such separate analyses based on CRC localization and stage could be important, because occurrence and severity of some side effects and symptoms are related to localization, stage and its resulting treatment, providing a possible explanation for decreased PA capacity among specific CRC subgroups. Examples are oxaliplatin-induced peripheral neuropathy, increased bowel dysfunction after radiation, and increased urogenital dysfunction among patients with a (prior) stoma (15).

Improved insight in potential determinants of PA among CRC patients during and after treatment is clinically relevant because it supports health care providers in providing individually tailored PA advice and guidance to improve long-term outcomes of CRC patients. This study therefore aims to examine longitudinal associations between sociodemographic and disease-related factors and PA levels from diagnosis up to 5 yr after diagnosis in different CRC subgroups based on tumor type and stage. In addition, PA levels and characteristics of patients will be compared with those of the general population to help identify patient-specific sociodemographic determinants.

## METHODS

### Study Population

Data from patients who participated in two large cohort studies were combined: the Prospective Dutch Colorectal Cancer (PLCRC) cohort (NCT02070146, ClinicalTrials.gov) and the COlorectal cancer Longitudinal Observational study on Nutritional and lifestyle factors that may influence colorectal tumor recurrence, survival, and quality of life—COLON cohort (NCT03191110, ClinicalTrials.gov).

#### PLCRC cohort

PLCRC is an ongoing prospective observational nationwide cohort in the Netherlands. All adult (≥18 yr) patients with a CRC diagnosis are eligible to participate. Patients can be recruited at any point in time, but preferably shortly after CRC diagnosis. As part of the informed consent procedure, patients are asked for consent to receiving repeated questionnaires at standardized intervals until 10 yr after first inclusion. Data from patients enrolled between February 2013 and October 2021 was used. PLCRC was approved by the Medical Research Ethics Committee of Utrecht, the Netherlands (METC 12-510). All patients signed informed consent. A detailed description of the cohort design can be found elsewhere (16).

#### COLON cohort

COLON is a prospective, observational cohort, for which newly diagnosed CRC patients were recruited in 11 hospitals in the Netherlands directly after diagnosis. CRC patients were recruited between August 2010 and February 2020. As part of the informed consent procedure, patients were asked for consent to receiving repeated questionnaires at standardized intervals until 5 yr after diagnosis. Non–Dutch-speaking patients, patients with a history of CRC, (partial) bowel resection, chronic inflammatory bowel disease, hereditary CRC syndromes (e.g., Lynch syndrome, Familial Adenomatous Polyposis, Peutz-Jegher), dementia or another mental condition obstructing participation were excluded from the study. The COLON study was approved by the Committee on Research involving Human Subjects, region Arnhem-Nijmegen, the Netherlands (2009-349). All patients signed informed consent. A detailed description of the cohort design has been published previously (17).

In addition to the inclusion criteria from both cohort studies, patients were included for this analysis when they had completed a questionnaire within 60 d of primary CRC diagnosis. This resulted in a final study population of 3001 (of 7307) PLCRC and 1909 (of 2128) COLON patients.

#### General population

Data from a representative sample of the Dutch population were obtained from health surveys of 2016 to 2019 conducted by Statistics Netherlands (CBS) (18–21). This annual survey collects information on demographics and health-related aspects in an annually renewed sample of >10,000 men and women of all ages. Physical activity was quantified using the same questionnaire as used in the two patient cohorts and the same syntax was used to calculate PA variables as for the patient population (see details below). Participants were matched 1:1 with the CRC cohorts on sex and a 10-yr age range, selecting participants closest in age to the CRC group. This approach aimed to enhance the comparability between the general population and the older, predominantly male CRC population.

### Assessment of Determinants

Data from the Netherlands Cancer Registry (NCR) (PLCRC) and from the Dutch ColoRectal Audit (DCRA) (COLON) were used to collect information on age, sex, primary tumor location, cancer stage, surgical resection, presence of stoma, systemic treatment, and radiotherapy. Netherlands Cancer Registry data are retrieved from electronic health records by trained data managers. For the DCRA, each participating hospital has one appointed surgeon who is responsible for registering the data, and the majority of surgeons record the data themselves (22). Information on disease status and treatment is available for the primary CRC diagnosis (i.e., first disease episode). Information on (treatment of) possible recurrence and/or progression of disease was not available for the majority of patients and therefore not used in current analyses. Patient-reported data included baseline educational attainment (low, i.e., elementary and lower vocational education finished, and above), having a partner (yes/no), and repeated measures for body mass index (BMI), smoking status (current, former, never), alcohol use (heavy drinking, defined as >14 units per week for females and >21 units per week for males, yes/no), stoma presence (current, prior, none), and health-related quality of life (HRQoL). Health-related quality of life was assessed using the validated European Organization for the Research and Treatment of Cancer Quality of Life Questionnaire-Core 30 (EORTC QLQ-C30, version 3.0) (23,24). This questionnaire consists of multiple subscales and is used to evaluate patients’ global health, daily functioning, and complaints of common symptoms. All questions use a four-point ordinal scale ranging from “not at all” to “very much.” Raw scores for multi-item scales were calculated by taking the average of the contributing items. Linear transformation was used to standardize the raw scores to scores ranging from zero to a hundred, where a higher score represents a higher level of functioning (“better”) or a higher level of symptoms (“worse”) (25).

Based on previous evidence demonstrating the effectiveness of exercise programs in reducing fatigue, anxiety, and depression symptoms, we hypothesized that these factors might also be associated with lower levels of PA (5). Consequently, we chose to focus on the four-item emotional functioning and three-item fatigue subscales in our analyses. Effect estimators of a 0.5 standard deviation (SD) increase will be presented (i.e., 12 for emotional functioning and 15 for fatigue) (26).

Because patient and treatment characteristics vary across CRC stages and types, three subsets were created to obtain more homogeneous study populations:

1. Stage I–III colon cancer (stage I–III CC)
2. Stage I–III rectal cancer (stage I–III RC)
3. Stage IV colorectal cancer (stage IV CRC)

#### Assessment of outcome

Physical activity levels in both patient cohorts were measured with the SQUASH questionnaire, which is a validated questionnaire to assess habitual PA (27). Completed questionnaires were quantified by assigning a Metabolic Equivalent of Task (MET) value to each activity, using the Ainsworth compendium of physical activity (28). Physical activity levels were assessed by quantifying total hours per week spent on several activities. To enhance comparability with existing literature, we established two PA outcome variables. In the main analysis, we evaluated Sport and Leisure time Moderate and Vigorous PA (MVPA-SL). In addition, we assessed adherence to the American College of Sports Medicine (ACSM) PA guidelines. This approach allows for a comprehensive examination of PA levels and improves consistency with previous studies. MVPA-SL comprises sport and leisure time activities of moderate (MET ≥3) and vigorous (MET ≥6) intensity. In the SQUASH, the following activities are assessed: leisure time bicycling, gardening, odd jobs and a maximum of four different sports. The ACSM PA guideline advises a minimum of 150 min·wk^−1^ moderate to vigorous PA and muscle-strengthening activities of at least moderate intensity at least twice a week, which is similar to the advice to the general population (5). Compared with MVPA-SL, this variable also includes heavy household activities, heavy occupational work and commuting by bike and by foot in our analyses. Because regular activities are also included and the total amount of days on which respondents perform one or more MVPA activities cannot be extracted from the SQUASH, adherence is met in our analyses when respondents perform at least 1 min of MVPA from seven different activities and/or moments during the week. This conservative approach is in accordance with SQUASH analyses as performed by the Dutch National Institute for Public health and the Environment (29).

In pooled analyses of cohort studies, both PA variables are associated with prolonged survival in multiple cancers, including colorectal cancer (30,31).

For the stage I-III disease analyses, all the common timepoints of the two patient cohorts were used, including assessments at diagnosis (T0), and 6 months (T6), 12 months (T12), 24 months (T24), and 60 months (T60) after diagnosis. In the COLON cohort, only patients receiving adjuvant chemotherapy were sent questionnaires at T12. Due to limited sample sizes at follow-up, only T0 and T6 were used to perform the stage IV analyses.

### Missing Data

Missing covariate data were handled using multiple imputation by chained equations under the Missingness At Random (MAR) assumption (32). The mice package (v 3.15) in *R* was used (33). Imputed longitudinal outcome data were not used, considering possible Missingness Not At Random (MNAR) reasons for discontinuation of follow-up, and imputed tumor stage, and location data were also not used to ensure correct patient subpopulations. One hundred imputed data sets through 20 iterations were created, which is by default more than the proposed minimum of data sets (the percentage of incomplete cases) (34). Trace plots were checked visually for convergence.

### Data Analysis

Two separate analyses were conducted for each of the three subsets of CRC patients. In the main analysis, linear mixed models were performed to assess the association of independent variables on MVPA-SL at each timepoint. Within-subject dependency was accounted for by assigning a random slope for time to each individual participant. All variables were added to the model as main effect and with a time interaction to estimate associations between independent variables and PA levels for each time point. Tumor stage was not included in the stage I–III disease subsets, to prevent multicollinearity with treatment variables (systemic therapy and radiotherapy). In this analysis, the “prior” category of stoma at any time after diagnosis was recategorized to “no,” because no patients had a prior stoma at diagnosis, resulting in modeling errors. Continuous variables were mean-centered for linear models to prevent fitting errors.

In the secondary analysis, determinants of PA guideline adherence among CRC patients were assessed for each timepoint separately by using binomial logistic regression models; binomial mixed models were not possible due to convergence errors. To prevent multicollinearity, tumor stage was used as an independent variable at T0, and replaced with treatment variables at follow-up for both stage I–III subsets. Radiotherapy is not part of standard care for stage I–III CC patients and was, therefore, not used as an independent variable in this subset. The vast majority of stage I–III CRC patients received a surgical resection of the primary tumor, which was therefore not used as an independent variable for either stage I–III subset.

Considering the multiple testing over time, sociodemographic determinants of both analyses will be discussed in more detail if they are consistently significantly associated with PA levels. We defined consistency as a significant association at a minimum of four out of five timepoints in the two stage I-III subsets. In addition, treatment-related variables (e.g., having a stoma) with a significant association with PA levels at T6 will also be discussed. This distinction was made under the hypothesis that treatment-related variables are not expected to show a stable association over time. Given the restricted timepoint selection (T0 and T6 only), all statistically significant associations with PA levels will be discussed for the stage IV CRC patient subset.

Finally, MVPA-SL levels at diagnosis of all CRC patients were compared with a sample of the general Dutch population. Possible differences in relevant determinants of PA levels for CRC patients at T0 and the general Dutch population were explored, by repeating regression models using the sociodemographic variables that were available in both study populations (i.e. age, sex, BMI, educational attainment, smoking status, alcohol use). Determinants of MVPA-SL were assessed using linear regression models and determinants of PA guideline adherence using binomial logistic regression models.

A *P* value <0.050 was considered statistically significant. SPSS version 26 and R version 4.2.1 were used.

## RESULTS

Sociodemographic and treatment-related characteristics at diagnosis (baseline) for the total study population and the three patient subgroups, i.e., stage I–III CC, stage I–III RC, and stage IV CRC, and demographics (where available) for the sample of the general Dutch population are shown in Table 1. The majority of patients were male (63%) and the median age was 66 (IQR, 59–73). Missing data on covariables at diagnosis ranged from 0% to 4.8% in the total study sample.

**TABLE 1:** Characteristics of colorectal cancer patients at diagnosis (stratified by stage and type of cancer) and the general Dutch population, matched on sex and a 5-yr age range.

	Patients				General Population
	Total	St. I-III CC	St. I-III RC	St. IV CRC	Total
	(*N* = 4910)	(*n* = 2905)	(*n* = 1459)	(*n* = 436)	(*n* = 4910)
Sex					
Male	3111 (63%)	1716 (59%)	1029 (71%)	288 (66%)	3111 (63%)
Female	1799 (37%)	1189 (41%)	430 (29%)	148 (34%)	1799 (37%)
Age					
Median (IQR)	66.0 (59.1, 73.0)	67.0 (61.0, 73.0)	65.0 (58.0, 72.0)	64.5 (57.0, 71.0)	66.0 (59.0, 73.0)
MVPA-SL					
Median (IQR)	5.0 (1.5, 10.5)	5.0 (1.5, 10.0)	5.0 (2.0, 11.0)	4.3 (1.0, 9.8)	6.0 (1.5, 12.5)
Missing	220 (4.5%)	117 (4.0%)	54 (3.7%)	25 (5.7%)	0 (0%)
ACSM PA guideline adherence					
No	2866 (61%)	1724 (62%)	819 (58%)	266 (65%)	2737 (56%)
Yes	1824 (39%)	1064 (38%)	586 (42%)	145 (35%)	2173 (44%)
Missing	220 (4.5%)	117 (4.0%)	54 (3.7%)	25 (5.7%)	0 (0%)
BMI					
Median (IQR)	26.0 (23.8, 28.7)	26.2 (23.8, 29.0)	25.9 (24.0, 28.7)	25.3 (23.2, 28.1)	25.9 (23.7, 28.7)
Missing	110 (2.2%)	58 (2.0%)	25 (1.7%)	6 (1.4%)	0 (0%)
Educational attainment*^a^*					
Low	1676 (36%)	1040 (37%)	471 (33%)	132 (32%)	1811 (37%)
Intermediate	1372 (29%)	804 (29%)	418 (30%)	123 (30%)	1810 (37%)
High	1661 (35%)	953 (34%)	521 (37%)	159 (38%)	1289 (26%)
Missing	201 (4.1%)	108 (3.7%)	49 (3.4%)	22 (5.0%)	0 (0%)
Heavy drinker*^b^*					
No	4261 (91%)	2522 (91%)	1275 (91%)	390 (95%)	4449 (91%)
Yes	418 (9%)	252 (9%)	133 (9%)	21 (5%)	461 (9%)
Missing	231 (4.7%)	131 (4.5%)	51 (3.5%)	25 (5.7%)	0 (0%)
Smoking status					
Current	387 (8%)	204 (7%)	140 (10%)	38 (9%)	863 (18%)
Former	2772 (59%)	1658 (59%)	825 (58%)	231 (56%)	2469 (50%)
Never	1568 (33%)	946 (34%)	454 (32%)	144 (35%)	1578 (32%)
Missing	183 (3.7%)	97 (3.3%)	40 (2.7%)	23 (5.3%)	0 (0%)
Marital status					
Married/common law	3888 (82%)	2288 (81%)	1181 (83%)	344 (83%)	—
Single (includes single with kids)	843 (18%)	522 (19%)	238 (17%)	71 (17%)	—
Missing	179 (3.6%)	95 (3.3%)	40 (2.7%)	21 (4.8%)	—
Surgical resection					
No	253 (5%)	6 (<0.1%)	98 (7%)	148 (35%)	—
Yes	4562 (95%)	2899 (100%)	1360 (93%)	274 (65%)	—
Missing	95 (1.9%)	0 (0%)	1 (0.1%)	14 (3.2%)	—
Stoma					
No	3637 (76%)	2720 (95%)	610 (42%)	284 (68%)	—
Yes	1128 (24%)	157 (5%)	835 (58%)	132 (32%)	—
Missing	145 (3.0%)	28 (1.0%)	14 (1.0%)	20 (4.6%)	—
Systemic therapy (any)*^c^*					
No	3062 (64%)	1921 (67%)	987 (68%)	128 (30%)	—
Yes	1737 (36%)	967 (33%)	467 (32%)	301 (70%)	—
Missing	111 (2.3%)	17 (0.6%)	5 (0.3%)	7 (1.6%)	—
Radiotherapy (any)					—
No	3787 (80%)	2833 (99%)	610 (42%)	317 (74%)	—
Yes	958 (20%)	16 (1%)	831 (58%)	110 (26%)	—
Missing	165 (3.4%)	56 (1.9%)	18 (1.2%)	9 (2.1%)	—
Fatigue score*^d^*					—
Median (IQR)	22 (0, 33)	22 (0, 33)	11 (0, 33)	33 (11, 44)	—
Missing	234 (4.8%)	132 (4.5%)	43 (2.9%)	25 (5.7%)	—
Emotional functioning score*^d^*					—
Median (IQR)	83 (667, 100)	83 (67, 100)	83 (67, 92)	83.3 (67, 92)	—
Missing	218 (4.4%)	117 (4.0%)	42 (2.9%)	25 (5.7%)	—

Missing percentages are presented as percentages from the total sample, but are not included in the non-missing counts.

*^a^*low education: elementary and lower vocational education, intermediate: secondary education, high: higher vocational and university education.

*^b^*Heavy drinking: more than 14 units per week for females and more than 21 units per week for males.

*^c^*Includes any chemotherapy administered, i.e. (neo-)adjuvant, in the context of chemoradiotherapy and palliative chemotherapy.

*^d^*Subscale of the EORTC core quality of life questionnaire (EORTC QLQ-C30.

*Abbreviations*:*; St. I-III CC*, stage I-III colon cancer; *St. I-III RC*, stage I-III rectal cancer; *st. IV CRC*, stage IV colorectal cancer; *IQR*, interquartile range; *ACSM*, American College of Sports Medicine; *PA*, physical activity*; MVPA-SL*, sport and leisure time moderate and vigorous physical activity; *U*, unit; *M*; male, *F*, female; *BMI*, body mass index.

Within the subgroup of stage I-III CC (*n* = 2905), 59% of patients were male, with a median age of 67 (IQR, 61–73). Regarding treatment, less than 1% did not undergo surgical resection, 5% received a stoma, and 33% received systemic therapy during their first disease episode.

Within the subgroup of stage I-III RC (*n* = 1459), 71% of patients were male, with a median age of 65 (58–72). Regarding treatment, 7% did not undergo surgical resection, 58% of patients received a stoma, 32% received systemic therapy, and 58% received radiotherapy during their first disease episode. Within the stage IV CRC subgroup (*n* = 436), 54% of patients were male, with a median age of 65 (57–71). Regarding treatment, 65% did not receive a surgical resection of the primary tumor, 32% of patients received a stoma, 70% received systemic therapy, 26% received radiotherapy during their first disease episode.

In comparison to the general Dutch population, CRC patients had a similar median BMI and similar proportions of low educational attainment and heavy drinking at the time of diagnosis. Fewer CRC patients were current smokers, but the proportion of never smokers was similar to the general Dutch population.

PA information was available for 4604 (T0), 3895 (T6), 2336 (T12), 2678 (T24), and 798 (T60) CRC patients. Note that numbers decrease over time partially because patients have not yet reached the later time points (2% at T6, 4% at T12, 17% at T24, and 58% at T60) or were deceased (1% at T6, 2% at T12, 4% at T24, and 6% at T60). Patients who filled in more than one questionnaire had higher MVPA-SL levels and a higher proportion PA guideline adherence, regardless of tumor stage and type (median hours per week MVPA-SL 5 vs 3 and adherence to PA guidelines 40% vs 27% in the total study sample, data not shown).

### MVPA-SL

**FIGURE 1:**
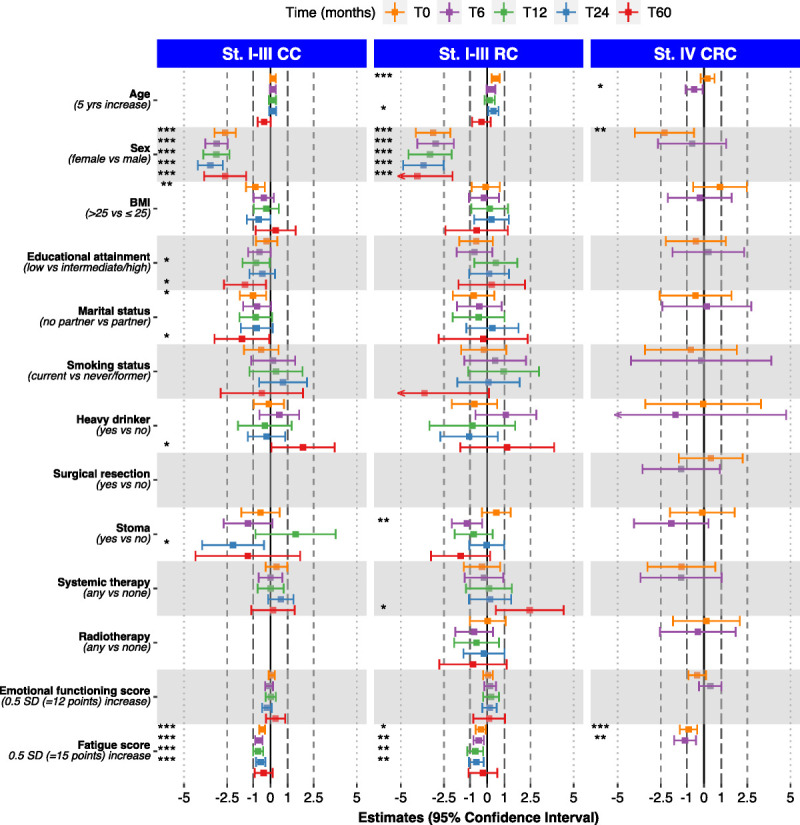
Determinants of hours per week Sport and Leisure time Moderate and Vigorous PA (MVPA-SL) for subgroups of CRC patients. *p = 0.01 to <0.05, **p = 0.001 to <0.01, and ***p < 0.001. T0, at diagnosis; T6, 6 months after diagnosis; T12, 12 months after diagnosis; T24, 24 months after diagnosis; T60, 60 months after diagnosis. St. I-III CC, stage I-III colon cancer; St. I-III RC, stage I-III rectal cancer; st. IV CRC, stage IV colorectal cancer.

An overview of determinants of MVPA-SL (hours per week) for the three patient subgroups over time is presented in Figure 1. Numerical estimates with 95% CIs can be found in Supplemental Tables 1a –1c, Supplemental Digital Content, http://links.lww.com/MSS/C973, Determinants of hours per week MVPA-SL among stage I-III colon cancer patients, stage I-III rectal cancer patients, and stage IV colorectal cancer patients).

For both stage I–III CC and stage I-III RC patients, female sex and higher fatigue scores were consistently significantly associated with lower levels of MVPA-SL. In addition, among stage I-III RC patients, having a stoma at T6 was significantly associated with lower PA levels. Among stage IV CRC patients, a higher fatigue score was significantly associated with lower levels of MVPA-SL at both T0 and T6. Female sex was significantly associated with lower PA levels at T0, as was a higher age at T6.

### ACSM PA guideline adherence

**FIGURE 2:**
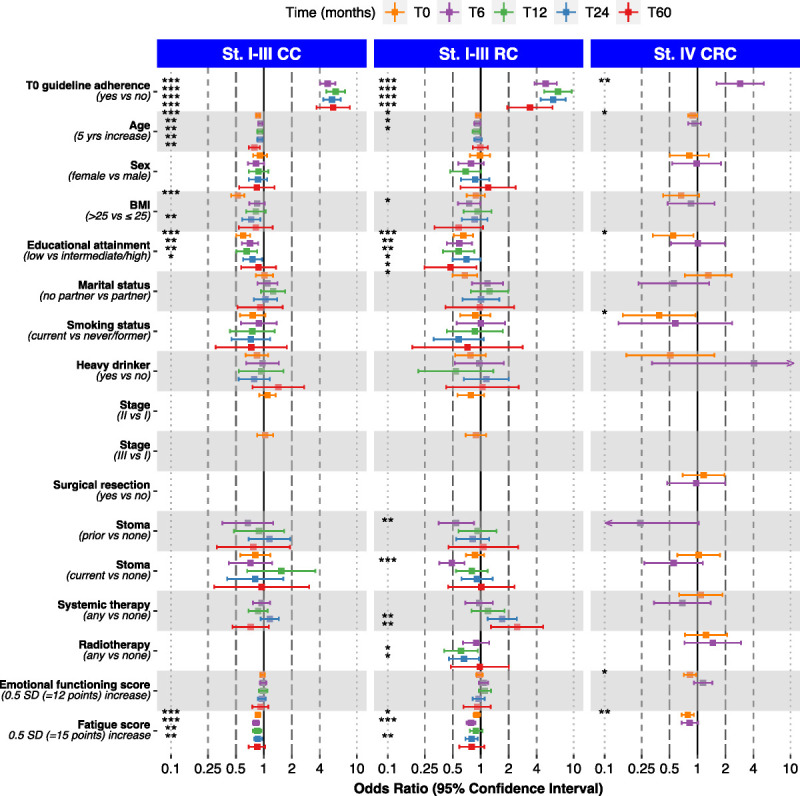
Determinants of ACSM PA guideline adherence (yes) for subgroups of CRC patients. *p = 0.01 to <0.05, **p = 0.001 to <0.01, and ***p < 0.001.

An overview of determinants of ACSM PA guideline adherence for the three patient subgroups over time is presented in Figure 2. Numerical ORs with 95% CIs can be found in Supplemental Tables 2a–2c (Supplemental Digital Content, http://links.lww.com/MSS/C973 Determinants of ACSM PA guideline adherence (yes) among stage I-III colon cancer patients, stage I-III rectal cancer patients, and stage IV colorectal cancer patients). For all CRC subgroups, PA guideline adherence at baseline was consistently significantly associated with a higher odds of PA guideline adherence at all timepoints after diagnosis. In addition, among stage I-III CC patients, higher fatigue scores, higher age and low educational attainment were consistently significantly associated with a lower odds of PA guideline adherence. Among stage I-III RC patients, low educational attainment was consistently associated with a lower odds of PA guideline adherence, and both a current and a prior stoma were significantly associated with a lower odds of PA guideline adherence at T6. Among stage IV CRC patients, higher age, low educational attainment, current smoking, higher emotional functioning and higher fatigue scores were significantly associated with a lower odds of PA guideline adherence at T0.

### Comparison with the general population

**FIGURE 3:**
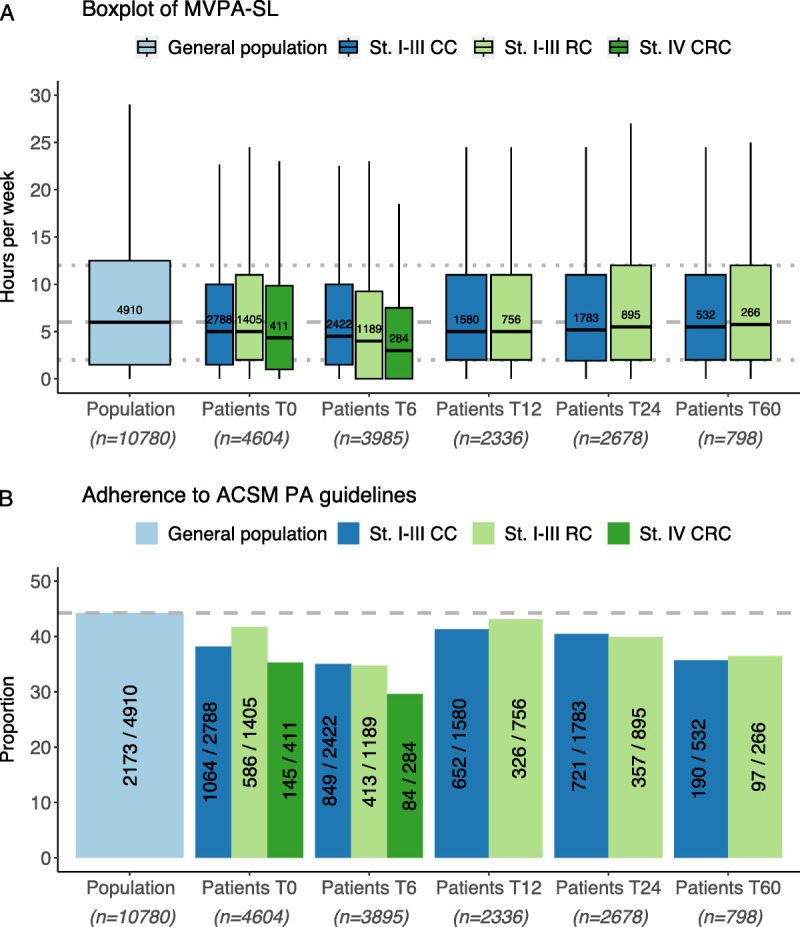
Physical activity levels of CRC patients and the general Dutch population, matched on age and sex.

An overview of hours per week MVPA-SL and of the proportion of adherence to ACSM PA guideline adherence for the general population and for CRC patients from diagnosis to 60 month post-diagnosis is presented in Figure 3. On average, all CRC patient subgroups were less active than the general population, although differences are small at timepoints T12 and further. The most notable difference was seen at T6 for both PA variables (median hours per week MVPA-SL 6 and 44% adherence to PA guidelines in the general population vs 3–4.5 and 30–35% for CRC patient subgroups).

**FIGURE 4:**
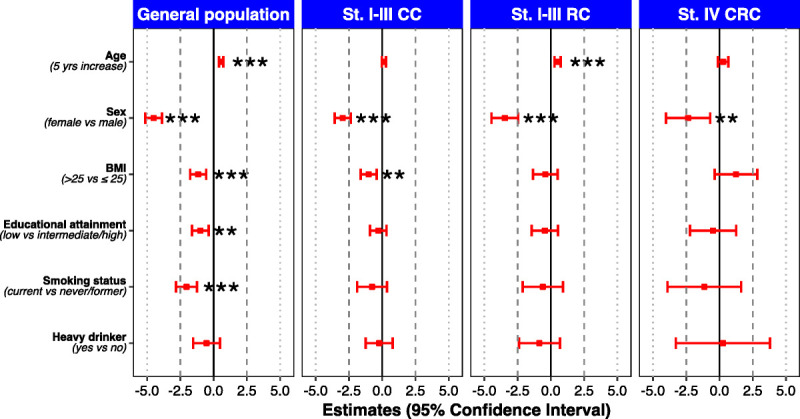
Determinants of hours per week MVPA-SL for subgroups of CRC patients at diagnosis, and for the general Dutch population, matched on age and sex. *p = 0.01 to <0.05, **p = 0.001 to <0.01, and ***p < 0.001.

An overview of the available sociodemographic determinants of MVPA-SL levels in both the sex- and age-matched general population and for the three CRC subgroups at diagnosis is presented in Figure 4. Numerical estimates and ORs with 95% CIs can be found in Supplemental Table 4 (Supplemental Digital Content http://links.lww.com/MSS/C973, Determinants of ACSM PA guideline adherence (yes) for subgroups of CRC patients at diagnosis, and for the general Dutch population, matched on age and sex). Among the general population, female sex, higher BMI, low educational attainment and current smoking were significantly associated with lower levels of MVPA-SL. Higher age was significantly associated with higher levels of MVPA-SL. Among all CRC subgroups, female sex was significantly associated with lower levels of MVPASL. In addition, higher BMI was significantly associated with lower levels of MVPASL among stage I–III CC patients and higher age was significantly associated with higher levels of MVPASL among stage I–III RC patients. The direction of association for all nonsignificant determinants is consistent with that observed in the general population, except for a higher BMI in the stage IV CRC subset.

**FIGURE 5:**
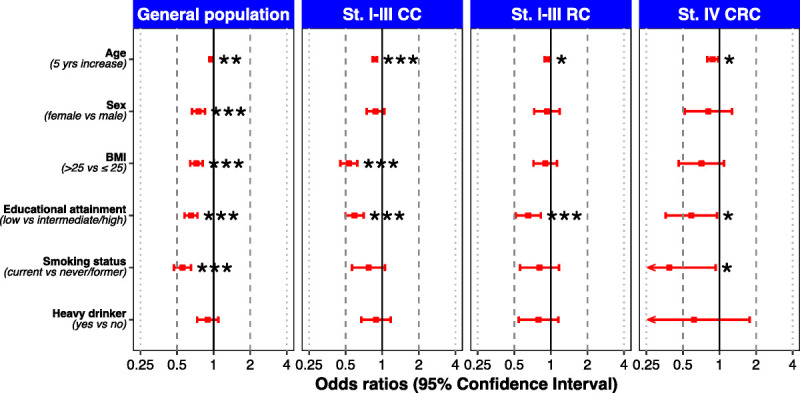
Determinants of ACSM PA guideline adherence (yes) for subgroups of CRC patients at diagnosis, and for the general Dutch population, matched on age and sex. *p = 0.01 to <0.05, **p = 0.001 to <0.01, and ***p < 0.001.

An overview of the available sociodemographic determinants of ACSM PA guideline adherence in both the sex- and age-matched general population and for the three CRC subgroups at diagnosis is presented in Figure 5. Numerical estimates and ORs with 95% CIs can be found in Supplemental Table 3 (Supplemental Digital Content, http://links.lww.com/MSS/C973). Among the general population, higher age, female sex, higher BMI, low educational attainment, and current smoking were significantly associated with a lower odds of PA guideline adherence. Among all CRC subgroups, higher age and low educational attainment were significantly associated with lower odds of PA guideline adherence. In addition, higher BMI was significantly associated with lower odds of PA guideline adherence among stage I–III CC patients and current smoking was significantly associated with lower odds of PA guideline adherence among stage IV CRC patients. The direction of association for all nonsignificant determinants is consistent with that observed in the general population.

## DISCUSSION

In this prospective observational cohort study, we found that higher fatigue scores and female sex were consistently significantly associated with lower MVPA-SL among all CRC patients over time. Physical activity guideline adherence at diagnosis was strongly and significantly associated with increased PA guideline adherence at all postdiagnosis timepoints among all CRC patients. Other notable findings were that of the disease-related determinants, only having a (prior) stoma was significantly associated with both lower levels of MVPA-SL and reduced PA guideline adherence among stage I–III RC patients. Systemic therapy and radiotherapy were not significantly associated with both MVPA-SL and PA guideline adherence among any of the CRC subgroups 6 months after diagnosis. Furthermore, we found that on average, all CRC patient subgroups were less physically active than the sex- and age-matched general population from diagnosis until 5 yr postdiagnosis, despite otherwise comparable demographics. The largest difference for all CRC subgroups was found at 6 months after diagnosis. Sociodemographic determinants of both MVPA-SL levels and PA guideline adherence were similar between CRC patients and the general Dutch population.

### Similarities and differences with other studies

Several studies previously investigated determinants of PA levels among CRC patients (9,13,14). Comparing determinants of PA from different studies poses multiple challenges due to heterogeneity in both assessment and categorization of PA. Studies used different PA questionnaires, resulting in varying ranges of MET-hours, possibly resulting in decreased comparability (27,35,36). The influence of PA categorization on results is illustrated by the different determinants we found for MVPA-SL and PA guideline adherence. Female sex, for example, showed a consistent association with lower MVPA-SL, but no association with PA guideline adherence, which also comprises moderate household and work activities, for all patient subgroups. This is in line with sex-stratified PA descriptives in our cohort, where the median time spent on MVPA-SL is very different, with 6 h·wk^−1^ for males and 3.5 for females, but the proportion PA guideline adherence is very similar (39% vs 38%). This implies that women with CRC on average spent less total time on MVPA-SL, but have sufficient PA levels in other domains to have similar PA guideline adherence proportions compared with men. This example emphasizes that associations differ depending on the selected physical activity variables.

For the MVPA-SL estimates, our analyses can be compared with Buffart et al. and van Putten et al. (9,13), who investigated linear associations with MVPA levels using the validated European Prospective Investigation into Cancer (EPIC) Physical Activity Questionnaire (35). The main difference with our study was that both other studies focused on long-term CRC survivors, where (first) questionnaires were sent out years after primary diagnosis, whereas we included patients starting from diagnosis onwards. The first approach might be limited by survival bias. In line with both studies, we also found that higher fatigue scores and female sex were consistently associated with lower MVPA-SL. Contrastingly, a higher BMI was not associated with lower MVPA-SL levels in our studies, compared with an observed significant association between a lower/healthy BMI and higher MVPA levels in both other studies. Focusing on disease-related determinants, we observed no association between tumor stage and PA levels which is similar to van Putten et al., but in contrast with Buffart et al. In addition, Buffart et al. reported that patients who were treated with chemotherapy had higher levels of MVPA. We also found a few significant associations between systemic therapy and higher MVPA-SL levels among stage I–III RC patients at later timepoints. This might be explained by a selection of patients who are fit for systemic therapy and have a good prognosis. Last, we observed a significant association between having a stoma and reduced PA levels among rectal cancer patients 6 months after diagnosis, but not at later timepoints. The results at later timepoints are in line with van Putten et al. who did not observe significant associations between having a stoma and MVPA years after diagnosis. A possible explanation of a temporary significant association shortly after treatment could be explained by increased coping over time with having a stoma (37,38).

For the PA guideline analyses, our results can be compared with Eyl et al. (14), who also performed a categorical analysis, but instead of PA guideline adherence used quartiles of MET-h/wk, with the first quartile being defined as physically inactive. Physical activity was quantified with the validated international physical activity questionnaire (IPAQ) (36) and measured around and 5 yr after diagnosis. Similar to their results, we also found reduced PA guideline adherence for higher age, and increased adherence when patients adhered to PA guidelines at baseline. Contrary to their findings, we found a clear association between low educational attainment and reduced PA guideline adherence, which was also observed in our analysis in the sample of the general Dutch population.

To our knowledge, no other study has yet compared PA levels of CRC patients to levels of the general population. It is important to acknowledge that the matching of the general population sample with the CRC study population based on age and sex introduces dependency in the data, which may lead to biased comparisons. Nevertheless, given the characteristics of the CRC population, specifically its older age group and male predominance, we considered it advantageous to conduct matching to ensure a fair comparison. We found that associations of the sociodemographic determinants age, sex, BMI, educational attainment, smoking status, and heavy drinking with PA levels did not differ substantially between the general population and CRC patients at diagnosis. Further, PA levels were generally lower in the CRC population, especially 6 months after diagnosis. This suggests that disease-related factors, such as treatment are responsible for this difference. Among these factors, fatigue emerged as the most important determinant in our data, as most treatment variables did not exhibit significant associations with PA levels in our analyses. This is an important finding as it has been demonstrated that physical exercise interventions can reduce fatigue among cancer patients and, hence, current international guidelines recommend exercise during and after treatment (5). Providing CRC patients with exercise guidance might help them break through this vicious cycle and reduce their fatigue symptoms by exercising more. An alternative explanation for the lower PA levels among CRC patients might be the association between low levels of physical activity and the elevated risk of developing CRC (39).

Our findings suggest that several sociodemographic and disease-related factors contribute to physical (in)activity. Increased knowledge of such factors is important to optimize targeted guidance of individual patients to help them increase their physical activity levels and thereby improve their well-being and possibly prognosis. Colorectal cancer patients will usually have consultations with health care professionals after diagnosis, providing an opportunity to ask them about their PA behavior and refer those in need to exercise specialists (40). The need for such guidance is illustrated by the fact that most CRC patients only marginally change their lifestyle after diagnosis (41). In addition, we observed a strong association between PA guideline adherence at diagnosis and adherence at follow-up. Our results comparing sociodemographic determinants of PA in both the general population and CRC patient seem to indicate that similar determinants are associated with physical inactivity. Future research focusing on determinants of CRC patients who changed their PA behavior from diagnosis onwards might help to further identify specific subgroups needing guidance the most. Including behavioral factors in such analyses likely provides further insight, since prior studies mentioned several behavioral factors such as intention to be physically active and beliefs about (adequate) physical activity (12,38,42,43). The same likely applies for including more (specific) psychological determinants. Chambers et al. studied the relationship over time between psychological distress and physical activity among CRC survivors. They reported associations between both somatization and anxiety with physical inactivity, besides also reporting associations between fatigue and physical inactivity (10). The absence of associations between PA levels and the EORTC QLQ-C30 emotional functioning subscale in our analyses might be explained by the fact that this subscale not purely focuses on anxiety, but instead covers aspects of anxiety, depression, and general distress.

### Strengths and Limitations

Our study has several strengths and limitations worth mentioning. This study is the largest to date using a nationwide combination of two CRC cohorts in which determinants of PA levels were assessed prospectively and longitudinally. Potential bias resulting from missing values was minimized by performing multiple imputation. Moreover, we were able to account for possible differences in disease-related characteristics by performing separate analyses for stage I-III colon and rectal cancer and for stage IV disease. Further, we repeated the analyses for two commonly used PA variables, thereby improving comparability with previous and future studies. In addition, we compared PA levels and the association between different sociodemographic factors and PA levels of CRC patients with the general population and found that the same determinants apply for both, which is important information for lifestyle programs that target the general population, including CRC patients.

Some limitations should also be considered. First of all, due to the observational design, causality could not be assessed. Second, because PA was self-reported, response bias may have occurred, so the absolute PA levels should be interpreted with caution. The exclusion of non-Dutch speaking and illiterate patients might have resulted in decreased generalizability to those subgroups. Physical activity guideline adherence is known to be lower among non-Western migrants compared with the native Dutch population (39.1 vs 50.7% in 2019) (21). Seeing how we compared our results mainly to two Dutch and one German studies, generalizability of our results to CRC patients in non-European countries is uncertain. The Dutch population is known to have the highest level of PA in Europe, and Americans are known to be much less physically active (44,45). However, this does not necessarily lead to different determinants of PA. For example, a prior study has shown that both PA levels and determinants of PA were different between White Europeans and south Asians recruited from primary care, but like us, they also reported significantly lower levels of MVPA for females (46).

An inherent limitation of MVPA-SL analyses is the lack of guidelines on a minimal clinically significant difference in MVPA-SL, which is why we focused on statistical significance. Although we were able to assess numerous determinants, not all potential determinants of PA levels were available, such as the beforementioned behavioral and social support factors, which would have helped further identifying subgroups. Information after initial cancer treatment was not available, meaning that changes in therapy possibly influencing PA levels, e.g., due to disease recurrence and/or progression, could not be accounted for. This is especially important for the stage IV CRC subgroup, which is a very heterogenous subgroup in both treatment and prognosis, which is another reason why we decided to focus on the first two timepoints in this subgroup. In addition, despite our large sample size, the number of patients at some later follow-up time points was still relatively small, especially in the stage IV CRC subset, resulting in large confidence intervals for our estimates. Response rates declined over time in our study for multiple reasons and may have introduced selection bias. Respondents who completed multiple questionnaires were, on average, more active compared with respondents who completed only one PA questionnaire, which could lead to an overestimation of PA levels postdiagnosis and an emphasis on determinants of PA among a relatively active CRC population.

## CONCLUSIONS

Female sex and higher fatigue scores were consistent determinants of lower MVPA-SL levels among all CRC patients over time, and PA levels were lowest at 6 months postdiagnosis. Sociodemographic determinants of PA were comparable with the general population. Our results can inform the design of intervention studies aimed at improving PA, and guide healthcare professionals in optimizing individualized support.
